# Intracellular sorting and transcytosis of the rat transferrin receptor antibody OX26 across the blood–brain barrier *in vitro* is dependent on its binding affinity

**DOI:** 10.1111/jnc.14482

**Published:** 2018-08-16

**Authors:** Arsalan S. Haqqani, George Thom, Matthew Burrell, Christie E. Delaney, Eric Brunette, Ewa Baumann, Caroline Sodja, Anna Jezierski, Carl Webster, Danica B. Stanimirovic

**Affiliations:** ^1^ National Research Council of Canada Human Health Therapeutics Research Centre Ottawa ON Canada; ^2^ Antibody Discovery and Protein Engineering MedImmune, Milstein Building Granta Park Cambridge UK

**Keywords:** affinity optimization, blood–brain barrier, intracellular trafficking, quantitative targeted proteomics, transferrin receptor antibody

## Abstract

The blood–brain barrier (BBB) is a formidable obstacle to the delivery of therapeutics to the brain. Antibodies that bind transferrin receptor (TfR), which is enriched in brain endothelial cells, have been shown to cross the BBB and are being developed as fusion proteins to deliver therapeutic cargos to brain targets. Various antibodies have been developed for this purpose and their *in vivo* evaluation demonstrated that either low affinity or monovalent receptor binding re‐directs their transcellular trafficking away from lysosomal degradation and toward improved exocytosis on the abluminal side of the BBB. However, these studies have been performed with antibodies that recognize different TfR epitopes and have different binding characteristics, preventing inter‐study comparisons. In this study, the efficiency of transcytosis *in vitro* and intracellular trafficking in endosomal compartments were evaluated in an *in vitro* BBB model for affinity variants (*K*_d_ from 5 to174 nM) of the rat TfR‐binding antibody, OX26. Distribution in subcellular fractions of the rat brain endothelial cells was determined using both targeted quantitative proteomics‐selected reaction monitoring and fluorescent imaging with markers of early‐ and late endosomes. The OX26 variants with affinities of 76 and 108 nM showed improved trancytosis (P_app_ values) across the *in vitro* BBB model compared with a 5 nM OX26. Although ~40% of the 5 nM OX26 and ~35% of TfR co‐localized with late‐endosome/lysosome compartment, 76 and 108 nM affinity variants showed lower amounts in lysosomes and a predominant co‐localization with early endosome markers. The study links bivalent TfR antibody affinity to mechanisms of sorting and trafficking away from late endosomes and lysosomes, resulting in improvement in their transcytosis efficiency.

**Open Practices:**

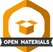

Open Science: This manuscript was awarded with the Open Materials Badge.

For more information see: https://cos.io/our-services/open-science-badges/

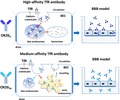

Cover Image for this issue: doi: 10.1111/jnc.14193.

**Abbreviations used**
ADAM: a disintegrin and metalloproteinase
BBB: blood–brain barrier
BEC: brain endothelial cells
BME: basal medium eagle
CDR: complementarity determining region
CHO: Chinese hamster ovary
DMEM: Dulbecco's modified Eagle's medium
EDTA: ethylenediaminetetraacetic acid
EEA1: early endosome antigen 1
HBSS: Hank's balanced salt solution
HDF: high‐density fractions
HRP: horseradish peroxidase
IgG: immunoglobulin G
LAMP1: lysosomal‐associated membrane protein 1
LDF: low‐density fractions
nanoLC MS/MS: Nano‐liquid chromatography mass spectrometry
PBS: physiological buffered saline
RAs: rat astrocytes
RFP: red fluorescent protein
RMT: receptor‐mediated transcytosis
RRID: research resource identifier
SDS‐PAGE: sodium dodecyl sulfate polyacrylamide gel electrophoresis
SNARE: soluble N‐ethylmaleimide‐sensitive factor activating protein receptor
SRM: selected reaction monitoring
SV‐ARBEC: SV40‐immortalized adult rat brain endothelial cells
TCEP: tris(2‐carboxyethyl)phosphine
TfR: transferrin receptor
UPLC: ultra performance liquid chromatography
VHDF: very high‐density fractions

Delivery of antibodies into the brain is highly restricted because of a tightly sealed layer of endothelial cells in brain microvessels that form the blood–brain barrier (BBB). Improved brain delivery of antibodies can be achieved via receptor‐mediated transport. A group of receptors expressed on the luminal surface of brain endothelial cells (BEC) are involved in constitutive or stimulated internalization, transport to the abluminal side and recycling, carrying and delivering ‘large’ protein ligands required for brain homeostasis, such as receptors for transferrin, insulin, insulin‐like growth factors or lipids (Lajoie and Shusta 2015). The effective delivery of pharmacologically active therapeutic payloads across the BBB can be achieved using antibodies that hijack the pathways mediated by these receptors (Lajoie and Shusta 2015). While the proof of concept of this approach has been demonstrated in rodents and non‐human primates, and is currently being tested in clinical trials, the understanding of mechanisms of transcytosis across the BEC remains sparse. The most studied receptor‐mediated transport receptor on the BBB is transferrin receptor (TfR). Studies with species‐selective mouse (Ri7; 8D3) (Manich *et al*. 2013; Cabezon *et al*. 2015) and rat (OX26) (Moos and Morgan 2001) antibodies binding TfR with high affinity, have yielded controversial results regarding their ability to efficiently transcytose the BBB and release into brain parenchyma. Most studies observed efficient brain vessel targeting and internalization of these antibodies, aided by TfR enrichment in BEC, but also their minimal ‘release’ into brain parenchyma. Recently, TfR antibodies re‐engineered in various antibody formats have been shown to more efficiently release into the brain parenchyma when their affinities are lowered or when the receptor is engaged by a monovalent antibody. In a recent study (Bien‐Ly *et al*. 2014), high‐affinity monovalent anti‐TfR antibodies increased TfR internalization and altered the trafficking patterns and fate of the receptor in BEC by inducing TfR movement toward lysosomal degradation; similarly, these anti‐TfR antibodies caused TfR degradation in the brain parenchyma, supporting the hypothesis that cellular TfR trafficking is altered from recycling to degradation because of high‐affinity anti‐TfR binding (Yu *et al*. 2011). Although similar results were reported by Niewoehner and co‐workers (Niewoehner *et al*. 2014), they argued that the recycling rate of TfR engaged with bivalent TfR antibody is defective and that monovalent mode of TfR binding enables its escape from lysosmal pathway and degradation, regardless of the receptor binding affinities. In a recent study, (Villaseñor *et al*. 2016) they showed a preferential sorting of a monovalent TfR antibody into sorting tubules which facilitated transcytosis across the BBB. However, a bivalent antibody that bound TfR in a pH‐sensitive fashion with lower affinity at acidic pH, typical of endosomal compartments, was found to escape degradation and release more efficiently on abluminal side of the BBB model *in vitro* (Sade *et al*. 2014). These studies were based on immunofluorescence co‐localization of antibodies with markers of early endosomes or lysosmes and were, at most, semiquantitative. Recently, (Thom *et al*. 2018), we found that lowering the affinity of OX26 from 5 nM to a range of 76–108 nM resulted in a > 50‐fold improved brain exposure over 96 h, because of both improved serum pharmacokinetics and higher transcytosis efficiency.

In this study, internalization and sorting of these OX26 affinity variants and TfR were evaluated in 20 subcellular fractions of rat BEC using quantitative, multiplexed mass spectrometry (SRM) methods in combination with immunofluorescence. Lowering the affinity of OX26 to 76–108 nM range resulted in a higher proportion of the antibody being sorted into high‐density subcellular fractions (HDF), which contained markers of early endosomes and recycling endosomes. The levels in HDF also matched the improvement in transcytosis across the BBB model *in vitro*. This study demonstrates that affinity engineering, in the absence of monovalent receptor binding, is sufficient to re‐direct TfR antibody intracellular trafficking away from lysosomes and to improve the efficiency of their transcytosis.

## Materials and methods

### Protein expression and purification

OX26 affinity variants were developed, expressed, and purified as described in detail previously (Thom *et al*. 2018). Briefly, DNA encoding the V_H_ and V_L_ of the mouse anti‐rat TfR antibody OX26 was synthesized by Life Technologies (Carlsbad, CA, USA) and cloned into expression vectors containing the appropriate light or heavy chain constant regions (Persic *et al*. 1997). Single alanine substitutions were introduced into HCDR1, HCDR3, or LCDR3 and the resulting mutants were characterized using an assay in which binding was monitored using the Octet RED384 System (Pall ForteBio LLC, Fremont, CA, USA) with anti‐hIgG capture Biosensors (18‐5060, Pall ForteBio LLC). An affinity of 5 nM was determined for wild‐type OX26 and the single alanine substitutions HCDR1 W33A, LCDR3 W96A, and HCDR3 F99A resulted in variant antibodies with *K*_D_ values of 76, 108, and 174 nM, respectively. These were then named as OX26 with a suffix denoting the affinity (OX26_5_, OX26_76_, OX26_108_, and OX26_174_) (Thom *et al*. 2018). Unless otherwise stated, OX26 variants and the control antibody, NiP228, an antibody against 4‐hydroxy‐3‐iodo‐5‐nitrophenylacetic acid (Webster *et al*. 2017)**,** were expressed as chimeric human IgG1 molecules with the S239D/A330L/I332E triple mutation (IgG1 TM) (Oganesyan *et al*. 2008). Antibodies were expressed in transiently transfected Chinese hamster ovary (CHO) cells in serum‐free media as described previously (Daramola *et al*. 2014). The concentration of IgG was determined by *A*_280_ using an extinction coefficient based on the amino acid sequence of the IgG (Pace *et al*. 1995). To allow site‐specific conjugation of fluorescent labels, antibodies were generated containing three cysteine residues introduced into the solvent‐exposed surface of the Fc region (Thompson *et al*. 2016). In some experiments, fusion of A20.1, a camelid single‐domain antibody against *C. difficile* toxin B, and the mouse Fc (A20.1mFc), also expressed in CHO cells, was used for normalization of responses across experimental groups.

### Rat brain endothelial cell line

An immortalized adult rat brain microvascular endothelial cell line, SV40‐immortalized adult rat brain endothelial cells (SV‐ARBEC) [(Muruganandam *et al*. 1997; Garberg *et al*. 2005); supplementary materials], was used for cellular internalization studies, *in vitro* transcytosis assays, and for endosome isolation and characterization. SV‐ARBEC cell line is not listed as commonly misidentified cell line by the ICLAC. The karyotype authentication of SV‐ARBEC was performed in 2003 prior to banking. Cells were banked at passage 76 and used in these studies between passage 78 and 86. The expression of rat‐specific genes/variants was confirmed using high‐throughput sequencing in 2017. SV‐ARBEC cells were grown in M199‐based feeding media (316‐010‐CL, Wisent, St‐Bruno, Quebec) containing: 0.25% Peptone (P‐5905), 0.9% d‐glucose (G‐8769), BME Amino Acids (B6766), BME Vitamins (B6891) – all from Sigma‐Aldrich (St. Louis, MO, USA); 10% heat‐inactivated fetal bovine serum (SH30396.03, Hyclone, Fisher Scientific, Ottawa, ON, USA) and antibiotic/antimycotic as previously described.

**Figure 1:**
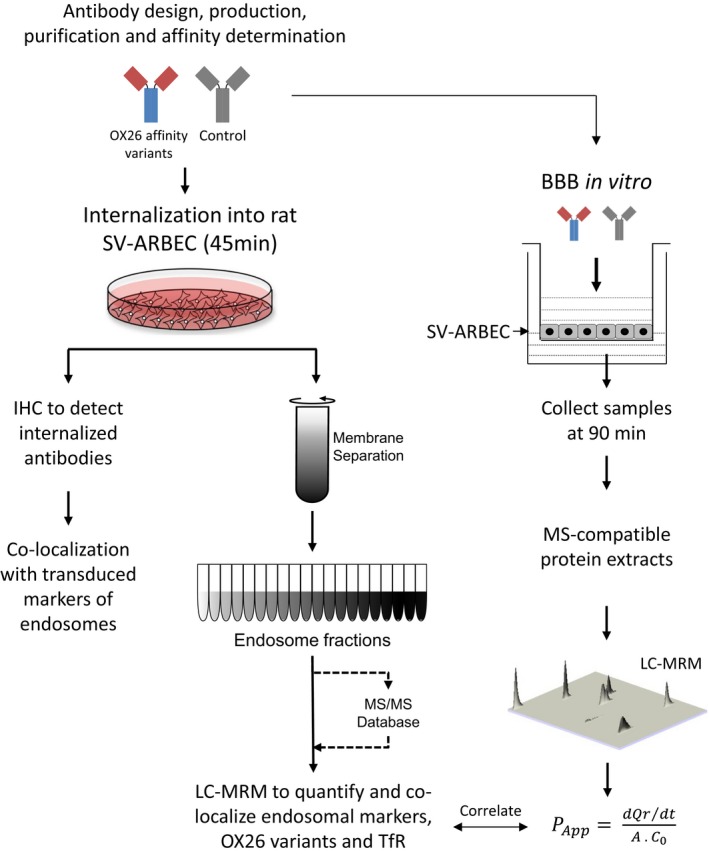
A schematic outlining the experimental design of the study. Antibodies were characterized for their internalization into rat BEC cell line, SV‐ARBEC, and their co‐localization with markers of early and late endosomes was determined using immunofluorescence methods. Cells were fractionated and each fraction was analyzed by nanoLC‐MRM to quantify the levels of: internalized antibodies, TfR, and markers of early and late endosomes. The antibodies were then evaluated for their ability to traverse SV‐ARBEC monolayer in transwells *in vitro*. Apparent peramebility coefficients (P_app_) were calculated for each antibody. Levels of the antibodies measured in early‐endosome‐ and late‐endosome/lysosome ‐containing compartments of SV‐ARBEC was then correlated with their P_app_ values.

The overall study design is schematically shown in Fig. 1.

### Western blot analyses

Cell lysates of SV‐ARBEC were prepared in RIPA buffer (50 mM Tris, pH 7.4, 150 mM NaCl, 2 mM ethylenediaminetetraacetic acid (EDTA), 0.1% sodium dodecyl sulfate (SDS), 1% Deoxycholate, 1% triton X‐100) containing protease inhibitor cocktail (11697498001, Roche, Laval, QC, USA). Proteins were separated by 8% sodium dodecyl sulfate polyacrylamide gel electrophoresis, transferred to nitrocellulose (162‐0115, Bio‐Rad, Mississauga, ON, USA), and probed with human‐rat‐cross reactive anti‐Transferrin Receptor antibody (13‐6800, RRID:AB_86623, Thermofisher Scientific, Nepean, ON, USA), followed by the horseradish peroxidase‐conjugated anti‐mouse secondary antibodies (315‐035‐045, RRID:AB_2340066, Jackson ImmunoResearch, West Grove, PA, USA). Blots were developed with Immunstar ECL kit (170‐5060, Bio‐Rad, Mississauga, ON, USA) and imaged on a FluorChem 8900 imager (Alpha Innotech, San Leandro, CA, USA). Images were processed using Adobe Photoshop (Adobe Systems Incorporated). For quantification, blots were stripped using 1 M Tris pH 6.8 (0497, Amresco, Solon, OH, USA), 2% SDS (L4509, Sigma Aldrich, St‐Louis, MO, USA), and 0.7% β‐mercaptoethanol (M7154, Sigma Aldrich, St‐Louis, MO, USA), and were re‐probed with β‐actin‐horseradish peroxidase antibodies (A3854, RRID:AB_262011,Sigma, Oakville, ON, USA).

### Antibody internalization into SV‐ARBEC cells

OX26 and control antibody variants containing engineered cysteines in their Fc region were labeled using Alexa 680 maleimide (A‐20344, Thermo Fisher Scientific, Rockford, IL, USA). The antibodies were reduced at 25°C for 2 h using a 40‐fold molar excess of tris(2‐carboxyethyl)phosphine (PG82089, Thermo Fisher Scientific) to generate free sulfhydryl groups (–SH). The tris(2‐carboxyethyl)phosphine was then removed using ZEBA desalting spin columns (87768, Thermo Fisher Scientific). The antibodies were then re‐oxidized with a 20‐fold molar excess of dehydroascorbic acid (dhAA) (262556, Sigma‐Aldrich) for 3.5 h at RT. The remaining free sulfhydryl groups (–SH) were reacted with a 10‐fold molar excess of AL680 maleimide for 1 h at RT followed by 24 h at 4°C. A four‐fold molar excess of N‐acetyl‐L‐cysteine (NAC) (A7250, Sigma‐Aldrich) was added for 1 h at RT at the end of the reaction to block any residual sulfhydryl. The antibodies were then purified using ZEBA desalting spin columns and further purified and concentrated using Amicon 4 (Ultracel‐30) spin columns (UFC803096, Millipore, Burlington, ON, USA). Protein concentration and dye to protein ratios were determined by measuring A_280_ and A_679_.

SV‐ARBEC cells (p83) were plated on a rat tail collagen I – (CACB354326 or 354236, BD Biosciences, San Jose, CA, USA) coated cover slips in a 24‐well plate and used for uptake studies at ~90% confluency. Cells were rinsed in 1× Hank's buffered saline solution (HBSS) (311‐513‐CL, Wisent, St‐Bruno, QC, USA) and 500 μL of cold Dulbecco's modified Eagle's medium (DMEM) (319‐005‐CL, Wisent, St‐Bruno, QC, USA) was added to each coverslip; cells were further kept on ice for 10 min. Cells were then incubated with a) 300 μL DMEM or b) 300 μL DMEM containing neutralized Al680 or c) 300 μL of each Al680‐labeled antibody in DMEM at 1.25 μM concentration for 15 min at 4°C. Cells were then washed 3× with cold DMEM, supplemented with 500 μL cold DMEM and incubated at 37°C for 45 min. At the end of incubation period, cells were washed with 1 mL cold DMEM, cover slips were fixed in 10% Formalin (SF‐100‐4, Thermofisher, Fair Lawn, NJ, USA) for 10 min at 25°C, washed again 2× in physiological buffered saline (PBS) (311‐010‐CL, Wisent, St‐Bruno, QC, USA) and stored in PBS at 4°C overnight. Coverslips were mounted in Dako Fluorescent Mounting Medium (S3023, Dako, Burlington, ON, USA) spiked with 2 μg/mL of Hoechst33342 (H3570, Life Sciences, Burlington, ON, USA) to stain cell nuclei and were then observed under Olympus 1 × 81 fluorescent microscope (40× oil objective, NA 1.42).

### BBB model *in vitro*

*In vitro* BBB permeability assays were performed using recently described protocols (Farrington *et al*. 2014; Webster *et al*. 2016). In brief, SV‐ARBEC were seeded at 80 000 cells/membrane on rat tail collagen coated 0.83 cm^2^ Falcon cell inserts, 1 μm pore size (353103, Corning, Durham, NC, USA) in 1 mL SV‐ARBEC feeding media without phenol red. The inserts were placed in the wells of a 12‐well tissue culture plate containing 2 mL of 50 : 50 (v/v) mixture of SV‐ARBEC feeding media without phenol red and rat astrocyte‐conditioned media to generate a model of the BBB *in vitro* as described previously (Garberg *et al*. 2005). Upon culturing, a barrier phenotype develops restricting the passage of molecules between chambers; permeability was monitored and the cultures used only when P_e_[sucrose] was between 0.4 and 0.6 [×10^−3^] cm/min. Transport experiments were performed as described previously (Haqqani *et al*. 2013a) by adding an equimolar mixture (1.25 μM) of antibodies to the top chamber and by collecting a 100 μL aliquot from the bottom chamber at 90 min for simultaneous quantification of both the antibodies using the multiplexed SRM method. In these studies, the media in the upper chamber contained 5% fetal bovine serum. The apparent permeability coefficient P_app_ was calculated as described previously (Artursson and Karlsson 1991).

### Endosome isolation

Endosome isolation and characterization was performed as described recently (Haqqani *et al*. 2018). SV‐ARBEC were grown to confluency on rat tail collagen type I‐coated plastic dishes, as described previously (Garberg *et al*. 2005). Four 150 mm confluent dishes of SV‐ARBECs were washed with HBSS and incubated separately with 0.3 μM of each OX26 variant for 45 min to trigger the receptor‐mediated transport pathway. At the end of incubation, cells were washed twice with HBSS and scraped in ice‐cold Buffer A (250 mM sucrose, 20 mM tricine, 1 mM EDTA) at 4°C. The suspension was homogenized using a loose Dounce homogenizer (20 strokes) on ice. The homogenate was centrifuged at 1000 *g* (Eppendorf 5417R) for 10 min at 4°C and the supernatant (post‐nuclear fraction) was transferred to a fresh tube. The pellet was re‐homogenized and re‐centrifuged, and the resulting supernatant added to post‐nuclear fraction. The fraction was overlaid on 23 mL of 30% Percoll (17‐0891‐02, GE Healthcare, Chicago, IL, USA), diluted in Buffer A and centrifuged at 84 000 *g* for 30 min at 4°C in Optima TLX ultracentrifuge with 60 Ti rotor (Beckman Coulter, Mississauga, ON, USA). Plasma membrane (opaque‐white top layer) was collected and transferred to a fresh ultracentrifuge tube, to which 1.84 mL of Buffer B (50% Optiprep, 250 mM sucrose, 120 mM tricine, 6 mM EDTA) and 0.16 mL of Buffer A was added. The layer was overlaid with 3.5 mL of 20% and 3.5 mL of 10% Optiprep. The gradient was centrifuged at 100 000 *g* (Beckman) for 90 min in a SW40 rotor at 4°C. The separation was split into top and bottom parts and transferred to separate tubes. Each one was mixed with 4 mL of Buffer B and overlaid with 2 mL of 5% Optiprep. The gradient was centrifuged at 100 000 *g* (Beckman) for 18 h in a SW40 rotor at 4°C. A total of 20 equal fractions were collected and prepared for mass spectrometry.

The enrichment of various molecular markers in isolated fractions was evaluated using western blot and mass spectrometry as recently described (Haqqani *et al*. 2018). These studies identified low‐density fractions 2‐4 (LDFs) as late endosomes and lysosomes, high‐density fractions 4‐7 (HDFs) as early and recycling endosomes, and very high‐density fractions 8‐10 (VHDFs) as a subset of multivesicular bodies. The same designations are used in this study.

### Sample preparation for mass spectrometry

Samples from *in vitro* BBB model and endosome preparations were further processed for subsequent analysis by nanoLC‐SRM. Briefly, pure variants and samples from *in vitro* BBB model were reduced, alkylated, and trypsin digested using the previously described method (Haqqani *et al*. 2008). For endosome preparation, a filtered‐aided sample preparation method was used to prepare the samples for mass spectrometry (Wiśniewski *et al*. 2009). Briefly, each sample was reduced in 3.5% SDS, 100 mM Tris‐HCl, 100 mM dithiothreitol by boiling for 10 min. A 6.6‐volume of urea solution (8M Urea, 100 mM Tris‐HCl, pH 8.5) was added to the sample and they were transferred to pre‐wetted Amicon Ultra‐4 (Ultracel‐30) spin columns and spun as per manufacturer's instructions. The proteins were washed three times with the urea solution, alkylated [10 mM iodoacetamide (I1149, Sigma‐Aldrich), 30–60 min at room 20°C in dark], and then washed four times with the urea solution and four times with 50 mM ammonium bicarbonate (A6141, Sigma Aldrich, St‐Louis, MO, USA). The samples were digested using trypsin at 37°C and the peptides were eluted for SRM analysis.

### Mass spectrometry and selected reaction monitoring (SRM)

OX26 affinity variants and control antibody levels in samples from *in vitro* BBB model and in endosomal cell fractions were trypsin digested (see above) and quantified using targeted nanolc ms/MS on nanoAcquity UPLC (Waters, Milford, MA, USA) coupled to ESI LTQ XL ETD mass spectrometer (ThermoFisher) as previously described (Haqqani *et al*. 2018). Briefly, the samples were injected onto a PepMap100, 5 μm 100 angstrom C18 trap (160454, ThermoFisher, Waltham, MA, USA) followed by eluting onto a 100 μm I.D. × 10 cm 1.7 μm BEH130C18 nanoLC column (186003546, Waters) using a gradient from 0% to 20% acetonitrile (in 0.1% formic acid) in 1 min, 20 to 46% in 60 min, and 46 to 95% in 1 min at a flow rate of 400 nL/min. Data were acquired on ions with mass/charge (m/z) values between 400 and 2000 with 1.0 s scan duration and 0.1 s interscan interval. To develop the SRM assay for proteins, samples (pure antibodies and endosome fractions) were first analyzed by nanoLC MS/MS using data‐dependent acquisition to identify ionizible peptides of antibodies and of known receptor‐mediated transport receptors and markers of early endosomes, late endosomes, and lysosomes. The spectra were validated and multiplexed methods were created for SRM analysis to perform targeted quantification of multiple proteins in each fraction. SRM analyses were carried out using these multiplexed methods and signatures described in Tables S1 and S2. For ILIS‐based quantification, isotopically heavy versions of the peptides were synthesized (New England Peptide LLC, Gardner, MA, USA) containing heavy C‐terminus K (+8 Da). SRM analyses were carried out as previously described (Haqqani *et al*. 2013a). Results were analyzed using Skyline software (version 3.7.0.10940, RRID:SCR_014080) freely available from MacCross Lab (University of Washington, WA, USA).

### Immunofluorescence

SV‐ARBEC cells were grown to semi‐confluence (60 000 cell/coverslip) on glass coverslips coated with the rat tail collagen I in a 24‐well plate for 2 days. Cells were then transduced overnight with 25 μL/coverslip (~40 particles/cell) of either BacMam 2.0 Early Endosomes‐RFP (Rab5‐RFP) (C10587) or Late Endosomes‐RFP (Rab7‐RFP) (C10589), or lysosomes (Lamp1‐RFP) (C10597) (all Life Sciences). Cells were washed two times in DMEM then incubated with neutralized near‐infrared fluorescent probe Cy5.5 (PA15604, Life Sciences) diluted in DMEM, or with 5 μg of various antibodies labeled with Cy5.5 at 37°C for 30 min. Cells were then washed three times in DMEM and two times in PBS. Coverslips were fixed in 4% formaldehyde in PBS for 10 min at 20°C, washed three times in PBS and permeabilized in 0.1% TritonX‐100 for 3 min. After washing in PBS, cells were stained with 1 : 2000 Alexa Fluor 488 Phalloidin (A12379, RRID:AB_2315147, Life Sciences) for 5 min at RT to label F‐actin filaments. After washing in PBS, coverslips were mounted in Dako Fluorescent Mounting Medium (S3023, Dako, Burlington, ON, USA) spiked with 2 μg/mL of Hoechst33342 (Life Sciences) to stain cell nuclei and were then observed under Olympus 1 × 81 fluorescent microscope (60× oil objective, NA 1.42).

### Statistical analyses

The personnel performing *in vitro* BBB assay studies and cellular uptake studies were blinded toward the ‘test articles’ used. Analytical measurements (multiplexed SRM) were performed by a separate organizational unit (Mass Spectrometry and Proteomics team) who were unblinded to experimental treatments. One‐way analysis of variance (anova) followed by Dunnett *post hoc* comparison of means was used to determine statistically significant differences between means of multiple independent groups (BBB‐crossing antibodies) against a control group (non‐BBB‐crossing antibodies). All statistical methods were carried out using GraphPad Prism^®^ 7.04 software. This study was not pre‐registered. The statistical methods to pre‐determine sample size were not employed, and no randomimzation methods were employed in this study.

## Results

### TfR expression in SV‐ARBEC cells and its co‐localization with markers of early and late endosomes

**Figure 2:**
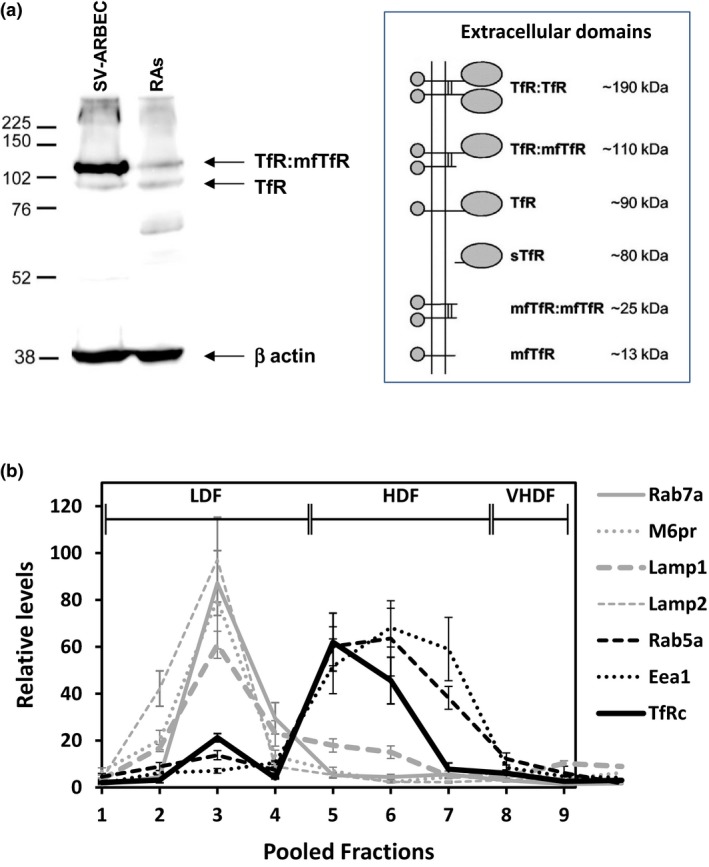
The expression and distribution of the transferrin receptor in cellular fractions of the immortalized rat brain endothelial cells (SV‐ARBEC). (a) Detection of the TfR by western blot in whole cell extracts of SV‐ARBEC and rat astrocytes (RAs) using pan‐specific rat‐human anti‐TfR antibody. The blot is representative of the *n* = 3 separate experiments. The schematic on the left hand side, adapted from (Kaup *et al*. 2002), shows different forms of the TfR detected in cells (sTfR is cleaved by membrane proteases; all other forms are membrane‐attached). Both SV‐ARBEC and RAs express TfR:mTfR (110kD) and TfR (90 kD) form of the receptor. (b) Relative levels of TfR, markers of early endosomes (Rab5a, EEA1) and markers of late endosomes (Rab7, Lamp1, Lamp2. M6pr) in cellular fractions of SV‐ARBECs quantified using multiplexed LC‐SRM. Shown are relative abundances (mean ± SD; *n* = 4 separate experiments) of protein‐specific peptides from three endosome preparations. Fractions 1‐4 are designated low‐density fractions (LDFs); fractions 5‐7 high‐density fractions (HDFs); fractions 8‐10 very high‐density fractions (vHDFs).

The TfR receptor expression in SV‐ARBEC cells, as well as in rat astrocytes (Rs) was analyzed by western blot (Fig. 2a) using a human‐rat cross‐reactive anti‐TfR antibody. TfR in the cell membrane appears in different mono‐ or homodimerized forms depicted in the schematic in Fig. 2a, adapted from (Kaup *et al*. 2002), because of proteolytic processing and shedding by various membrane proteases, including A disintegrin and metalloproteinase (Kaup *et al*. 2002). Altogether six TfR fragments were detected and identified by size and immunological characteristics as follows: ∼190‐kDa dimer of TfR (TfR:TfR), ∼110‐kDa dimer of TfR lacking one extracellular domain (TfR:mfTfR), ∼90‐kDa monomeric TfR (TfR), ∼80‐kDa soluble monomeric TfR (sTfR), ∼25‐kDa dimer of TfR lacking both extracellular domains (mfTfR:mfTfR), and ∼13‐kDa monomeric membrane fragment (mfTfR). The prevalence of these forms in different cell types is variable; in SV‐ARBEC used for these studies, the forms with one extracellular domain were dominant (Fig. 2a); the band at ~110kD corresponds to the TfR:mfTfR, whereas the lower band (~90kD) corresponds to the monomeric TfR as described by Kaup (Kaup *et al*. 2002). In some cultures, an apparent full dimeric receptor was detectable at MW ~210 kD (data not shown).

TfR:mfTfR form showed much higher levels by western blot in SV‐ARBEC cells compared to RAs (Fig. 2a), consistent with the known enrichment of TfR in rat BEC (Enerson and Drewes 2006).

Next, we evaluated TfR distribution in various SV‐ARBEC intracellular compartments. Recently, we have described gradient fractionation and quantitative protein characterization of subcellular fractions of SV‐ARBEC using nanoLC‐SRM (Haqqani *et al*. 2018). The low‐density fractions (LDF 2‐4) contained markers of late endosomes/lysosomes (Lamp 1/2, rM6pr, Rab7a, Rab11a/b), whereas high‐density fractions (HDF 5‐7) were enriched in markers of early and recycling endosomes (Rab5a, Eaa1) [(Haqqani *et al*. 2018); Table 3]; very high‐density fractions (VHDF 8‐10) exhibited unique profile of markers, similar to some subpopulations of microvesicular bodies (Haqqani *et al*. 2018). In SV‐ARBEC cultured in transferrin‐containing media (TIBC‐235 μg/dL) TfR was distributed 25:75% between LDFs and HDFs (Fig. 2b). The late endosome and lysosome markers (Rab 7, M6Pr, and LAMPs) peaked in LDF fractions 2‐4, whereas early and recycling endosome markers (EEA and Rab 5) peaked in HDFs late endosome fractions 5‐8 (Fig. 2b, Table S3). An expanded list of proteins enriched in LDFs and HDFs in SV‐ARBECs under basal conditions is shown in Table S3. Notably, clathrin, vesicle‐soluble N‐ethylmaleimide‐sensitive factor activating protein receptor (SNARE) family member cellubrevin (VAMP3), low‐density lipoprotein‐related protein‐1 (LRP‐1), insulin receptor, insulin growth factor 1 receptor (IGF1R), the membrane P4‐ATPase flippase ATP8b1 and its β‐subunit TMEM30A, previously identified as target for the BBB‐crossing single‐domain antibody FC5, were all enriched in HDF fractions (Table S3 ). LDFs were enriched in caveolin‐1, flotilin‐1, vesicle‐SNARE member synaptobrevin (VAMP family) and its interacting protein synaptosomal‐associated protein 23 (SNAP23), both essential components of the general membrane fusion machinery and important regulators of transport vesicle docking and fusion (Table S3). Interestingly, known receptor‐mediated transcytosis receptors, including TfR, were all enriched in HDF fractions (Table S3).

### OX26 affinity variant internalization and transcytosis across SV‐ARBEC cells

**Figure 3:**
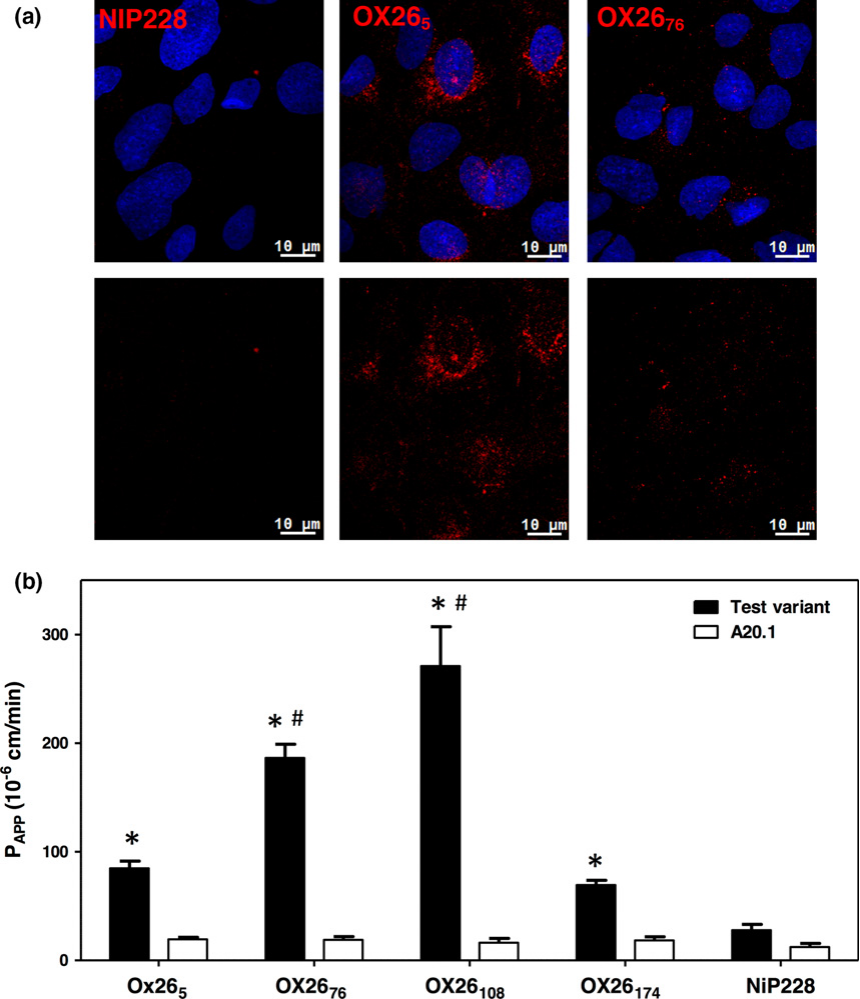
Internalization and transcytosis of OX26 antibody affinity variants in rat model of the blood–brain barrier (BBB) *in vitro*. (a) SV‐ARBEC cells were exposed to fluorescently labeled control antibody NiP228, or OX26_5_, or OX26_76_ for 45 min and internalization of the antibody was assessed by fluorescent microscopy. Fluorescent images in upper panels are fusion of red (antibody) and blue channels (cell nuclei counter‐stained by Hoechst); bottom images show red signal of the antibody. (b) Apparent permeability coefficient (P_app_) of OX26 affinity variants and the control antibody NiP228 in SV‐ARBEC BBB model *in vitro*. Single‐domain antibody A20.1 was used in each transwell insert as an ‘in‐experiment’ control for the monolayer permeability. Results are shown as Mean ± SD for *n* = 6 independent transwell inserts. Asterisks (*) indicate *p *<* *0.01 compared to NiP228; number signs (#) indicate *p *<* *0.01 compared to OX26_5_ (one‐way anova followed by Dunnet's *post hoc* comparison of means).

The binding of OX26 affinity variants to SV‐ARBEC as well as enhanced brain exposure of lower affinity variants has been reported recently (Thom *et al*. 2018). In this study, we examined the internalization and intracellular distribution of these variants. Both fluorescently labeled OX26_5_ and OX26_76_ showed strong internalization into SV‐ARBEC (Fig. 3a), in contrast to no appreciable internalization of the control antibody NiP228 (Fig. 3a). Although OX26_5_ was detected in large vesicles surrounding cell nuclei (Fig. 3a), lower affinity OX26_76_ showed more diffuse distribution within cells (Fig. 3a). Internalization of OX26_108_ was similar to that of OX26_76_, whereas OX26_174_ showed significantly reduced internalization compared with other variants (fluorescence data not shown – see quantitative levels in the subsequent section).

The rate of transcytosis of OX26 affinity variants was examined in a BBB model *in vitro* formed by SV‐ARBEC cells as described previously (Garberg *et al*. 2005; Farrington *et al*. 2014; Webster *et al*. 2016). A single domain antibody against C. difficile toxin B, A20.1 (17kD) (Hussack *et al*. 2011) was added together with each ‘test antibody’ into top compartments and levels of antibodies transmigrated across endothelial monolayer were measured after 90 min using multiplexed SRM. A20.1 and the control IgG, NiP228, showed low levels in bottom chambers in each measurement (Fig. 3b). Given that A20.1 and NiP228 do not bind mammalian receptors, their minimal crossing of the BEC monolayer may be because of either low paracellular migration or non‐specific pinocytosis or both. In contrast, OX26_5_, OX26_76_, OX26_108_, and OX26_174_ levels in the bottom compartments of the model were 4‐fold, 9‐fold, 13‐fold and 2.5‐fold higher, respectively, from those of co‐administered A20.1 or from that of the control IgG, NiP228 (Fig. 3b). Affinity variants OX26_76_ and OX26_108_ showed ~twofold and ~threefold higher P_app_ values, respectively, compared to high‐affinity OX26_5_ variant, whereas OX26_174_ P_app_ was lower than that of OX26_5_. The high levels of OX26 variants crossing the BBB model *in vitro* were interpreted as receptor‐mediated (transcellular) transcytosis, in contrast to low levels of non‐specific transport of control A20.1 and NiP228 antibodies.

### OX26 affinity variants sorting in subcellular compartments of SV‐ARBEC cells

SV‐ARBEC cells exposed to 0.3 μM of OX26 affinity variants for 45 min were fractionated as described (Haqqani *et al*. 2018) and levels of antibody variants, TfR, and markers of early (EEA1, Rab5) and late (Rab7, M6Pr, LAMP1) endosomes determined in each fraction using multiplex SRM method. In separate experiments, the internalization and co‐localization of OX26 variants with early and late endosomes were determined in SV‐ARBEC transfected with RFP‐labeled Rab5a and Lamp1, respectively, using immunofluorescent detection.

**Figure 4:**
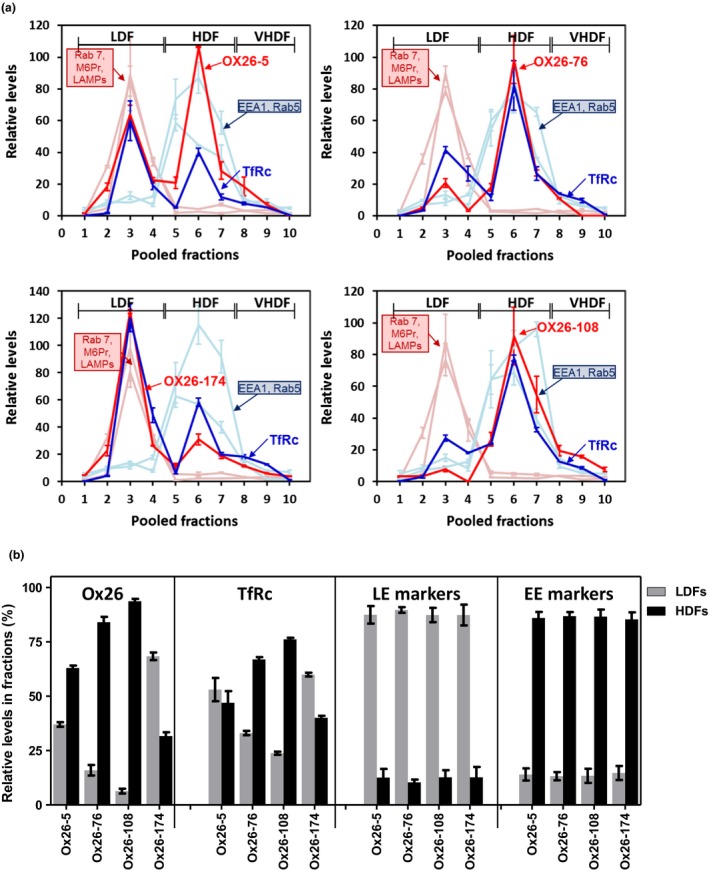
Co‐localization of OX26 affinity variants and TfR with markers of early and late endosomes/lysosomes in subcellular fractions of SV‐ARBEC cells. (a) Cells were exposed to 0.3 μM of either one of OX26 affinity variants for 45 min, fractionated and analyzed by multiplexed LC‐SRM. Graphs show relative levels of the OX26 variant (solid black lines), TfR (dashed black lines), markers of early endosomes (Rab5a, Eea1) (dashed gray lines) and markers of late endosomes (Rab7, Lamp1, Lamp2) (solid gray lines) in each cellular fraction. Fractions 1‐4 are designated low‐density fractions (LDFs); fractions 4‐8 high‐density fractions (HDFs); fractions 8‐10 very high‐density fractions (vHDFs). For OX26 variants, absolute levels were measured (using calibration curve and ILIS), whereas for other proteins only relative intensities were measured. Since MS intensities cannot be compared among different proteins but intensities of a same protein can be compared among different samples (fractions), all intensities were normalized to a constant total intensity and overlaid to allow comparison of relative levels of different proteins among different fractions. Shown are average intensities (± SD) of protein‐specific peptides from three biologically independent endosome preparations. Absolute levels of internalized OX26 antibodies were as follows: OX26_5_: 67.2 ± 3.1 amol; OX26_76_: 25.9 ± 4.7 amol; OX26_108_: 26.3 ± 2.1 amol; OX26_174_: 9.48 ± 1.8 amol. (b) Bar graph showing composite relative abundance (AUC; mean ± SD from n = 3 independent experiments/endosome preparations) of OX26 affinity variants, TfR and markers of late and early endosomes in LDFs and HDFs in each experimental condition shown in A. For ‘OX26’ and ‘TfRc’ panels, asterisks (*) indicate *p* < 0.01 compared to OX26_5_ LDFs; number signs (#) indicate *p* < 0.01 compared to OX26_5_ HDFs; ampersand (&) indicate *p* < 0.05 compared to OX26_5_ HDFs (one‐way anova followed by Dunnett *post hoc* comparison of means). For the ‘LE markers’ and ‘EE markers’ panels, asterisks (*) indicate *p* < 0.01 compared to respective LDFs (one‐way anova followed by Dunnett *post hoc* comparison of means).

High‐affinity OX26_5_ variant showed the highest internalization into SV‐ARBECs (total intracellular levels: 67.2 ± 3.1 amol) and distributed in LDF and HDF fractions at 38:62 ratio (Fig. 4a and b). LDF:HDF distribution of TfR in OX26_5_‐exposed cells was 65 : 35 (Fig. 4 a and b), a significant re‐distribution into late‐endosome/lysosome‐containing LDF fractions compared to its ‘constitutive’ distribution into HDFs (Fig. 2b). While OX26_76_ showed lower internalization into SV‐ARBEC (25.9 ± 4.7 amol) compared with OX26_5_, its intracellular distribution was high into HDF (LDF:HDF 15:85) and was accompanied with a predominant distribution of TfR into HDFs (LDF:HDF 30:70) (Fig. 4a and b). OX26_108_ (total internalized levels: 26.3 ± 2.1 amol) exhibited further increase in HDFs (LDF:HDF 6:94) with TfR partitioning 25:75 between LDF and HDFs (Fig. 4a and b). A small amount (9.48 ± 1.8 amol) of OX26_174_ that internalized into SV‐ARBEC, partitioned highly into LDFs (LDF:HDF 70 : 30), with TfR showing a similar re‐distribution toward LDFs (LDF:HDF 60 : 40) (Fig. 4a and b). The distribution of early and late endosome markers among LDFs and HDFs in SV‐ARBECs was similar under basal conditions (Fig. 2b) and among OX26 variant‐stimulated conditions (Fig. 4a and b).

**Figure 5:**
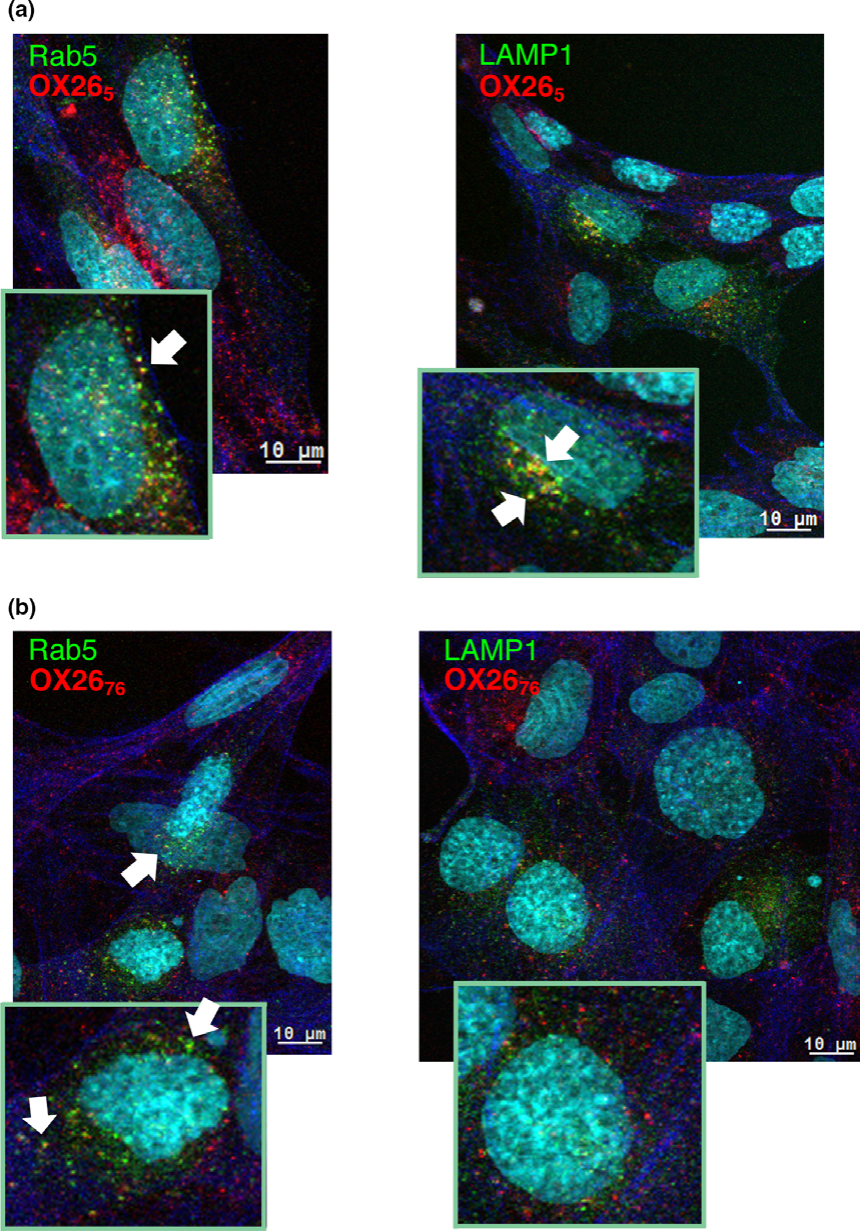
Co‐localization of AF680‐labeled (red) OX26_5_ (a) and OX26_76_ (b) with endosome markers in RFP‐Rab5 (left panels) and RFP‐Lamp‐1 (right panels) (both in green) – transduced SV‐ARBEC. Actin filaments labeled with Alexa Fluor 488 Phalloidin are shown in blue. Nuclei are labeled with Hoechst (shown in turquoise). Cells were transduced and internalization studies performed as described in Materials and methods. Micrographs are representative of *n* = 3 independent experiments.

Studies of OX26_5_ and OX26_76_ internalization and co‐localization with Rab5a and Lamp‐1 in Rab5a‐RFP or Lamp‐1‐RFP transfected SV‐ARBEC cells (Fig. 5) confirmed the findings obtained in fractionated SV‐ARBEC by multiplexed nanoLC‐SRM. While OX26_5_ co‐localized with both Rab5‐RFP and Lamp‐1‐RFP (Fig. 5a), OX26_76_ co‐localized with Rab5‐RFP only (Fig. 5b) and no co‐localization was detected with Lamp1‐RFP.

**Figure 6:**
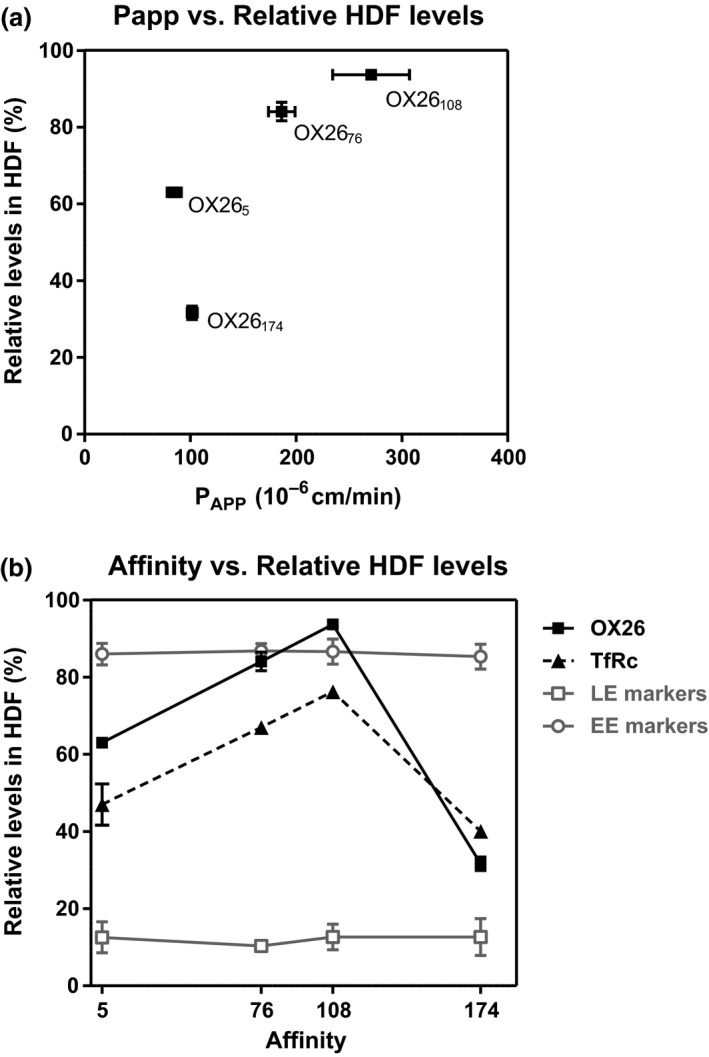
Relationship between OX26 variant binding affinities, their distribution in HDFs (early endosomes) and transcytosis across the blood–brain barrier (BBB) model *in vitro*. (a) P_app_ values versus percent distribution into HDFs of OX26 affinity variants. (b) Affinity versus percent distribution into HDFs of OX26 affinity variants. The relative distribution of the TfR, as well as early endosome (EE) and late endosome (LE) markers in cells exposed to each OX26 affinity variant is also shown.

Overall, in the affinity range of 5‐108 nM, the proportion of OX26 affinity variants partitioning into early endosome‐containing HDFs was inversely correlated with their ability to transcytose the BBB model *in vitro* (P_app_) (Fig. 6a). However, lowering the affinity further, resulted in the intracellular partitioning of OX26_174_ into LDFs, similar to that observed with the high‐affinity OX26_5_ (Fig. 6b), although their internalized levels were vastly different. The data suggest a tight ‘window’ of optimal affinities at which the engagement of TfR with antibodies results in the traffic of receptor antibody complex similar to that observed with its natural ligand.

### TfR expression in SV‐ARBEC after exposure to OX26 affinity variants

**Figure 7:**
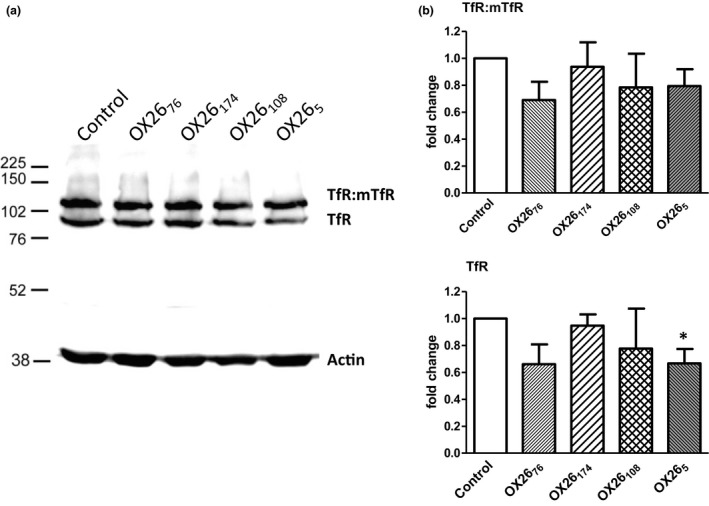
TfR levels in SV‐ARBEC after a 48‐h exposure to OX26 affinity variants. The TfR expression levels were determined by western blot as described in Materials and methods. (a) Gels shown are representative of three separate experiments. (b) Relative densities of each TfR‐specific band versus loading control β‐actin were determined and shown as mean ± SD (*n* = 3 separate western blots). Asterisks indicate a significant difference (*p* < 0.01, one‐way anova followed by Dunnett *pot hoc* comparison among means) compared to a corresponding band in cells under basal condition.

We examined whether the endosomal traffic of TfR triggered by various OX26 affinity variants may cause down‐regulation of TfR because of degradation. SV‐ARBECs were exposed to various OX26 affinity variants (0.3 μM) for 48 h, and TfR levels were evaluated by western blot (Fig. 7a). The only statistically significant change observed in this experiment was lowering the expression of TfR band (but not TfR:mTfR band) by OX26_5_ (Fig. 7b). The down‐regulation ‘trend’ of both TfR:mTfR and TfR expression was also seen with OX26_76_ and OX26_108_, although these changes did not reach statistical significance (Fig. 7a and b). OX26_174_ did not affect the expression levels of either TfR form in SV‐ARBEC (Fig. 7 A&B).

## Discussion

The findings of this study demonstrate that the intracellular distribution and endosomal sorting of TfR and TfR‐binding OX26 antibodies in rat BEC are affinity‐dependent and influence the antibody release on the abluminal side of the BBB model *in vitro*. The BBB transcytosis was inversely dependent on the proportion of the internalized antibody being sorted into late endosomes and lysosomes.

The transferrin receptor (TfR) is a type II transmembrane protein that mediates uptake of iron by binding the iron carrier protein transferrin (Tf). The 90 kDa TfR comprises a short cytoplasmic tail with an internalization motif, a membrane‐spanning portion, a stalk region which contains two disulfide bonds, which covalently link the two TfR monomers, and a large extracellular ectodomain (Feelders *et al*. 1999). Binding of human Tf (hTf) to TfR triggers conformational changes in the TfR (Eckenroth *et al*. 2011), which initiates its internalization. Following internalization of the complex, iron is released in the acidic endosomes and the TfR·Tf complex recycles back to the cell surface where apotransferrin is released at neutral pH. The TfR is highly expressed in brain endothelial cells and neurons (Jefferies *et al*. 1984), as well as in peripheral tissues, notably lung, liver, and reticulocytes (Chan and Gerhardt 1992).

Antibodies against TfR are being developed for delivery of therapeutics across the BBB; to avoid interference with the natural process of hTf/iron traffic into the brain, they should be raised to recognize epitopes in the extracellular domain of TfR away from Tf binding sites. TfR antibodies are internalized into BEC via a clathrin‐dependent receptor‐mediated endocytosis (Qian *et al*. 2002); at present, it is not clear whether (at least some) antibodies could trigger TfR internalization in the absence of Tf binding to the receptor. The mechanisms of their subsequent intracellular sorting and abluminal exocytosis are subject to current debate. Recent studies have shown that the TfR antibody binding affinity (Yu *et al*. 2011; Thom *et al*. 2018), pH‐dependency of binding (Sade *et al*. 2014), and valency (Niewoehner *et al*. 2014) can all affect the efficiency of antibody release on the abluminal side of the BBB. Reduced TfR antibody affinity in general (Yu *et al*. 2011), or at acidic pH of late endosomes (Sade *et al*. 2014), is postulated to facilitate its dissociation from TfR and abluminal release; while cross‐linking of TfR by high‐affinity bivalent antibodies, in contrast to monovalent antibodies, was shown to drive the complex into degradative pathway (Niewoehner *et al*. 2014). Although not systematically studied, the epitope on TfR engaged by various antibodies is likely an additional important attribute that determines the nature of receptor engagement and their intracellular fate.

In this study, bivalent TfR antibody OX26 affinity variants with a conserved binding epitope were used to study internalization and intracellular co‐localization with TfR and multiple markers of endosomal compartments in rat BEC using multiplexed quantitative mass‐spectrometry methods. These methods allowed a more precise molecular characterization of intracellular/endosomal fractions containing internalized antibodies. For example, in addition to markers of early endosomes, Rab5a and EEA1, other known receptor‐mediated transport receptors, insulin receptor (IR), insulin‐like growth factor 1 receptor (IGF1R), and LRP1 were ‘tracked’ to a clathrin‐containing high‐density cellular fractions (HDFs); in contrast, late endosome/lysosome markers Lamp1, M6Pr, and Rab7a were found in low‐density cellular fractions (LDFs), also enriched with Rab11a, flotillins, caveolin‐1, and several other SNARE family proteins involved in the targeting of and/or fusion of transport vesicles to their target membrane.

The amount of OX26 affinity variants internalized into SV‐ARBECs was inversely proportional to their affinity. Internalized medium affinity OX26_76_ and OX26_108_, along with the TfR, distributed predominantly into HDFs containing, among others, markers of early/recycling endosomes, whereas high‐affinity OX26_5_ and low‐affinity OX26_174_, along with TfR, were both routed into LDFs, containing markers of late endosomes and lysosomes. OX26 affinity variants routed into HDFs showed an efficient release on the abluminal side of the rat BBB model *in vitro*; overall the transcytosis of variants across the BBB model *in vitro* correlated well with the proportion of antibodies sorted into early endosome fractions. A similar observation was reported by Bien‐Ly and colleagues (Bien‐Ly *et al*. 2014) with heterodimerized mouse‐specific TfR‐BACE1 bi‐specific antibodies; however, the affinity range of TfR antibody arm (K_D_‐250–600 nM) at which the BBB transcytosis was enhanced and intracellular traffic was routed through early endosomes was different from our study, likely because the antibodies were monovalent and were binding different TfR epitope(s) from those used in our study. The ‘window’ of binding affinities at which the bivalent OX26 antibodies preferentially trafficked to early/recycling endosomes and exhibited enhanced BBB transcytosis was relatively narrow (76‐108 nM) and lowering the affinity further (to 174 nM) resulted in small amount of internalized antibody being trafficked in a pattern similar to that of the high‐affinity (~5 nM) variant.

The molecular mechanisms that determine routing of the TfR/Ab complex into endosomal subcompartments are not fully understood. For example, based on molecular modeling, Niewoehner *et al*., (Niewoehner *et al*. 2014) argued that the bivalent TfR antibodies will cause cross‐linking of the TfR, triggering its trafficking to lysosomes where the complex is destined for degradation; in contrast, a monovalent TfR antibody, regardless of its affinity, will engage TfR without cross‐linking resulting in its efficient transcytosis across the BBB. A recent study (Villaseñor *et al*. 2016) implicated Rab17‐dependent sorting microtubules in directing monovalent TfR antibody toward successful transcytosis; the bivalent TfR antibody‐TfR clusters were excluded from sorting microtubules by slower diffusion and were targeted for degradation. This hypothesis did not take into account the presence of various forms of the TfR on the surface of cells, some of which are naturally dimerized; nor was a range of affinities tested in a monovalent format.

The extracellular domain of TfR is cleaved by membrane proteases to create a soluble monomeric TfR (sTfR) (Kaup *et al*. 2002), shed into serum as a free molecule or within exosomal particles (Ahn and Johnstone 1993). TfR has been detected previously in extracellular microvesicles generated by SV‐ARBEC (Haqqani *et al*. 2013b). Shedding of sTfR leaves a variety of membrane‐inserted forms of TfR including homodimer (~190kD depending on glycosylation pattern), homodimer with one extracellular domain (TfR:mfTfR; 110 kD), monomeric TfR (90 kD), as well as their respective 25kD and 13kD ‘headless’ fragments (Kaup *et al*. 2002). The prevalence of these forms in different cell types is variable; in rat BEC used for these studies, the monomeric TfR forms were dominant. It is likely that various fully or partially cleaved membrane TfR forms are also present in endothelial cells *in vivo*, since circulatory sTfR (reference values 1.8–4.6 mg/L) is used as diagnostic marker for iron status.

OX26 antibody variants will bind all membrane TfR forms that have intact (non‐cleaved) extracellular domain, and will likely trigger internalization regardless of whether they bind mono‐ or bivalent (homodimerized) TfR; however, their subsequent trafficking through intracellular compartments may be different. It is not clear whether the affinity of the binding antibody may determine preferential interactions with any specific form of TfR containing an extracellular domain. Since all TfR antibodies used in these studies were bivalent, they likely triggered some cross‐linking of various TfR forms expressing extracellular domain.

Studies by Bien‐Ly (Bien‐Ly *et al*. 2014) and Niewoehner (Niewoehner *et al*. 2014) both showed that TfR antibody formats that directed the complex to lysosmes may cause subsequent proteolytic degradation and down‐regulation of TfR in both BBB endothelial cells and brain tissue. Similarly, longer exposure to OX26_5_, OX26_76_, and OX26_108_ in this study caused an observable trend of TfR down‐regulation, with the monomeric TfR in cells exposed to OX26_5_ being significantly reduced. The data agree with measured proportion of TfR routed into LDFs by these variants. Although the high proportion of low‐affinity OX26_174_‐TfR complex was directed into late endosomes/lysosmes, the overall amount of internalized complex destined for degradation was low, and likely not sufficient to affect TfR levels over a 48 h exposure.

In a recent study, we have shown that lower affinity rat TfR antibody variants, OX26_76_ and OX26_108_, exhibited a 50‐fold enhanced brain exposure after systemic administration compared to a high‐affinity OX26_5_. In addition, a pharmacodynamic response to the analgesic peptide cargo chemically conjugated to these OX26 affinity variants was significantly enhanced with OX26_76_ and OX26_108_, compared to OX26_5_ (Thom *et al*. 2018). *In vitro* and the *in vivo* BBB transcytosis of the same OX26 affinity variants was in good agreement between this study and the *in vivo* study by Thom and co‐workers (Thom *et al*. 2018). The brain exposure of OX26 affinity variants *in vivo* was affected by both their serum pharmacokinetics and efficiency of transcytosis across the BBB (Thom *et al*. 2018). OX26_5_ exhibited a short plasma half‐life (6.1 h), was also mostly ‘trapped’ in brain vessels and did not produce appreciable staining of TfR‐expressing neurons. In contrast, OX26_76_ and OX26_108_ showed prolonged serum pharmacokinetics (~50 h) and were detected in both brain vessels and bound to neurons in the brain parenchyma. This study provides further evidence that the improved brain exposure of OX26_76_ and OX26_108_ *in vivo* was influenced by a more efficient process of transcytosis whereby the antibody undergoes preferential sorting into endosomal compartments destined for exocytosis. Despite displaying long serum half‐life, OX26_174_ produced a minimal brain exposure (Thom *et al*. 2018) because of both suboptimal affinity for receptor engagement *in vivo* and antibody traffic to the degradative pathway shown in this study. Affinity modulation of bivalent TfR antibodies was sufficient to impact the efficiency of BBB transcytosis in both studies.

Recent demonstrations that the transport efficiency of TfR antibodies across the BBB can be improved by various antibody engineering techniques rekindled the interest in their development as potential delivery vehicles for therapeutics targeting the CNS. Because antibodies used in different studies were species‐specific and recognized different epitopes on the TfR, it was difficult to generalize key observations as universal principles useful for antibody engineering to improve their BBB transport. The common evidence from this study and other available literature suggest that efficient transcytosis across the BBB could be achieved by engineering key receptor‐mediated transport antibody attributes that support its preferential routing into early endosomes and away from degradative pathways in BEC. These key attributes are likely receptor‐ligand specific and may include antibody affinity, its ability to trigger conformational changes and endocytosis of the receptor, the receptor epitope and the manner in which it is engaged (e.g., monovalent, bi‐paratopic, etc.), the pH dependence of antibody binding and intracellular routing signals engineered into the antibody.

## Open science badges

This article has received a badge for *Open Materials* because it provided all relevant information to reproduce the study in the manuscript. The complete Open Science Disclosure form for this article can be found at the end of the article. More information about the Open Practices badges can be found at https://cos.io/our-services/open-science-badges/.

## Supporting information


**Table S1**. Peptide ‘signature’ used for nanoLC‐SRM analysis of early endosome and late endosome/lysosome markers.
**Table S2**. Peptides used for nanoLC‐SRM detection of antibody variants used in the study.
**Table S3**. Relative abundance of various proteins involved in intracellular trafficking and receptor‐mediated transcytosis among low‐density (LDFs; fractions 2‐5) and high‐density (HDFs; fractions 4‐7) fractions in SV‐ARBEC cells exposed to under basal conditions.
