# Supplementary material for: Intracellular sorting and transcytosis of the rat transferrin receptor antibody OX26 across the blood–brain barrier in vitro is dependent on its binding affinity
Source: J Neurochem. 2018 Aug 16;146(6):735–52. doi: 10.1111/jnc.14482 (PMC6175443; doi:10.1111/jnc.14482)
Supplement: Supplementary file 1 — Table S1. Peptide ‘signature’ used for nanoLC‐SRM analysis of early endosome and late endosome/lysosome markers. Table S2. Peptides used for nanoLC‐SRM detection of antibody variants used in the study. Table S3. Relative abundance of various proteins involved in intracellular trafficking and receptor‐mediated transcytosis among low‐density (LDFs; fractions 2‐5) and high‐density (HDFs; fractions 4‐7) fractions in SV‐ARBEC cells exposed to under basal conditions. [file JNC-146-735-s001.pdf]

# Intracellular sorting and transcytosis of the rat transferrin receptor antibody OX26 across the blood-brain barrier *in vitro* is dependent on its binding affinity

Arsalan S. Haqqani[1], George Thom[2], Matthew Burrell[2], Christie E. Delaney[1], Eric Brunette[1], Ewa Baumann[1], Caroline Sodja[1], Anna Jezierski[1], Carl Webster[2], Danica B. Stanimirovic[1]

Affiliations:

[1]National Research Council of Canada, Human Health Therapeutics Research Centre, Ottawa, ON K1A0R6

[2]Antibody Discovery and Protein Engineering, MedImmune, Milstein Building, Granta Park. Cambridge. CB21 6GH. UK

Corresponding Author:

* Carl Webster, MedImmune, Milstein Building, Granta Park, Cambridge. CB21 6GH. United Kingdom email:websterc@medimmune.com, Tel: + 44 2037496652. Fax: +44 1223 471472

## Supplementary files

**Supplementary Table 1**. Peptide ‘signature’ used for nanoLC-SRM analysis of early endosome and late endosome/lysosome markers*

| Protein | Type | Peptide Sequence | Charge | m/z of peptide | Peptide fragment signatures (m/z) used for quantification |
|---|---|---|---|---|---|
| Early endosome antigen 1 (Eea1) | Early endosome marker | EAQNDLEQVLR | 2 | 657.84 | 515.33, 540.36, 644.37, 1009.46 |
| | | ELVQVQTLMDNMTLER | 2 | 960.48 | 878.4, 942.95, 1009.47, 1223.58 |
| | | KLDNTTAAVQELGR | 2 | 758.41 | 749.9, 772.43, 843.47, 944.52 |
| Ras-related protein Rab5a | Early endosome marker | AVDFQEAQSYADDNSLLFMETSAK | 2 | 1340.61 | 813.38, 1323.14, 1470.68, 1542.07 |
| | | FEIWDTAGQER | 2 | 676.32 | 489.24, 776.35, 962.44, 1075.52 |
| | | LVLLGESAVGK | 2 | 543.33 | 439.33, 647.34, 760.42, 873.5 |
| Ras-related protein Rab7a | Late endosome marker | DPENFPFVVLGNK | 2 | 738.38 | 431.26, 603.24, 873.52, 1020.59 |
| | | EAINVEQAFQTIAR | 2 | 795.42 | 795.52, 934.53, 1063.58, 1276.69 |
| | | LVTMQIWDTAGQER | 2 | 824.41 | 454.13, 489.24, 566.3, 962.43 |
| Ras-related protein Rab11a and b | Late endosome marker | AQIWDTAGQER | 2 | 637.81 | 503.17, 529.51, 962.6, 1075.67 |
| | | GAVGALLVYDIAK | 2 | 645.38 | 609.57, 708.7, 817.75, 973.76 |
| | | STIGVEFATR | 2 | 540.79 | 446.93, 522.94, 531.41, 779.41 |
| | | NNLSFIETSALDSTNVEEAFK | 2 | 1165.06 | 1024.49, 1139.52, 1147.54, 1252.61 |
| Cation-dependent mannose-6-phosphate receptor (M6pr) | Late endosome marker | GVGDDQLGEESEER | 2 | 760.33 | 649.28, 742.8, 835.35, 948.43 |
| | | RAVVMISCNR | 2 | 603.32 | 557.32, 585.8, 594.8, 1049.52 |
| | | SFESTVGQGSDTYSYIFR | 2 | 1022.47 | 685.37, 1004.91, 1208.56, 1393.67 |
| Lysosome-associated membrane glycoproteins Lamp1 and Lamp2 | Lysosome marker | ALQASVGNSYK | 2 | 569.30 | 569.3, 754.38, 825.42, 953.48 |
| | | GPDTVDSTTDIK | 2 | 624.80 | 624.89, 779.46, 878.46, 1094.54 |
| | | YLDFIFAVK | 2 | 558.31 | 249.16, 277.15, 724.44, 839.47 |
| Ras-related protein | MVB marker | FLALGDSGVGK | 2 | 532.29 | 803.66, 619.34, 522.24 |

| | | | | | |
|---|---|---|---|---|---|
| Rab27a | | | | | |
| Programmed cell death 6-interacting protein Pdcd6ip | MVB marker | LANQAADYFGDAFK | 2 | 765.86 | 1033.58, 1104.64, 962.78 |
| | | LLDEEEATDNDLR | 2 | 766.85 | 748.74, 733.73, 803.58 |
| | | NIQVSHQEFSK | 2 | 658.83 | 536.7, 649.59, 862.59 |
| CD82 antigen | MVB marker | EEAWDYVQAQVK | 2 | 733.35 | 950.42, 573.31, 875.05 |
| Transferrin receptor protein 1 (TfR) | RMT receptor | DMGLSLQWLYSAR | 2 | 770.38 | 496.25, 795.41, 1044.52, 1123.59 |
| | | LFWADLK | 2 | 446.75 | 343.02, 431.09, 446.75, 632.35 |
| | | SAFSNLFGGEPLSYTR | 2 | 873.43 | 736.41, 979.48, 1126.55 |

* Shown are the transitions for nanoLC-SRM detection/quantitation of various endocytic markers, receptors, $V_H$Hs, and $V_H$H-Fc fusion used in the study. In various experiments, analyses were multiplexed in different combinations for simultaneous monitoring of multiple peptides in the same sample.

**Supplementary Table 2**. Peptides used for nanoLC-SRM detection of antibody variants used in the study. In various studies, analyses were multiplexed in different combinations for simultaneous monitoring of multiple peptides in the same sample.

| Protein | Type | Peptide Sequence | Charge | m/z of peptide | Peptide fragment signatures (m/z) used for quantification |
|---|---|---|---|---|---|
| $OX26_5$ | Anti-TfR IgG 840 | TTPPVLDSDGSFFLYSK | 2 | 937.28 | 836.8, 1150.24, 1265.44, 1378.72, 1477.44 |
| | | TTPPVLDSDGSFFLYSK[(a)] | 2 | 941.28 | 840.5, 1158.25, 1273.44, 1386.74, 1485.44 |
| | | TVAAPSVFIFPPSDEQLK | 2 | 973.52 | 913.49, 1015.38, 1060.51 |
| | | SGTASVVCLLNNFYPR | 2 | 899.45 | 810.39, 435.24, 272.17 |
| $OX26_{76}$ | Anti-TfR IgG 819 | TTPPVLDSDGSFFLYSK | 2 | 937.28 | 836.8, 1150.24, 1265.44, 1378.72, 1477.44 |
| | | TTPPVLDSDGSFFLYSK[(a)] | 2 | 941.28 | 840.5, 1158.25, 1273.44, 1386.74, 1485.44 |
| | | TVAAPSVFIFPPSDEQLK | 2 | 973.52 | 913.49, 1015.38, 1060.51 |
| | | SGTASVVCLLNNFYPR | 2 | 899.45 | 810.39, 435.24, 272.17 |
| $OX26_{108}$ | Anti-TfR IgG 834 | TTPPVLDSDGSFFLYSK | 2 | 937.28 | 836.8, 1150.24, 1265.44, 1378.72, 1477.44 |
| | | TTPPVLDSDGSFFLYSK[(a)] | 2 | 941.28 | 840.5, 1158.25, 1273.44, 1386.74, 1485.44 |
| | | TVAAPSVFIFPPSDEQLK | 2 | 973.52 | 913.49, 1015.38, 1060.51 |
| | | SGTASVVCLLNNFYPR | 2 | 899.45 | 810.39, 435.24, 272.17 |
| $OX26_{174}$ | Anti-TfR IgG 822 | TTPPVLDSDGSFFLYSK | 2 | 937.28 | 836.8, 1150.24, 1265.44, 1378.72, 1477.44 |
| | | TTPPVLDSDGSFFLYSK[(a)] | 2 | 941.28 | 840.5, 1158.25, 1273.44, 1386.74, 1485.44 |
| | | TVAAPSVFIFPPSDEQLK | 2 | 973.52 | 913.49, 1015.38, 1060.51 |
| | | SGTASVVCLLNNFYPR | 2 | 899.45 | 810.39, 435.24, 272.17 |
| Nip228 | Control IgG | TTPPVLDSDGSFFLYSK | 2 | 937.28 | 836.8, 1150.24, 1265.44, 1378.72, 1477.44 |
| | | TTPPVLDSDGSFFLYSK[(a)] | 2 | 941.28 | 840.5, 1158.25, 1273.44, 1386.74, 1485.44 |
| | | TVAAPSVFIFPPSDEQLK | 2 | 973.52 | 913.49, 1015.38, 1060.51 |
| | | GTTVTVSSASTK | 2 | 569.74 | 780.32, 560.79, 679.3 |

| | | | | | |
|---|---|---|---|---|---|
| | | SGTASVVCLLNNFYPR | 2 | 899.45 | 810.39, 435.24, 272.17 |
| A20.1 | VHH, llama immune (C. diff toxin A) | TFSMDPMAWFR | 2 | 694.77 | 582.22, 807.4, 922.42, 1140.5 |
| | | TTYYADSVK | 2 | 524.23 | 203.1, 682.34, 845.4 |
| | | EFVAAGSSTGR | 2 | 541.29 | 500.12, 564.32, 635.28, 706.44 |
| | | DEYAYWGQGTQVTVSSGQAGQGSEQK | 2 | 1381.47 | 1061.0, 1163.5, 1580.4, 1598.5, 1748.8 |

(a) Isotopically labeled internal standards (ILIS)

**Supplementary Table 3**. Relative abundance of various proteins involved in intracellular trafficking and receptor-mediated transcytosis among low-density (LDFs; fractions 2-5) and high-density (HDFs; fractions 4-7) fractions in SV-ARBEC cells exposed to under basal conditions.

| | Symbol | LDF | HDF | Function |
|---|---|---|---|---|
| Phospholipid-transporting ATPase IC | Atp8b1 | 8% | 92% | Catalytic component of a P4-ATPase flippase complex which catalyzes the hydrolysis of ATP coupled to the transport of aminophospholipids from the outer to the inner leaflet of various membranes and ensures the maintenance of asymmetric distribution of phospholipids. Phospholipid translocation seems also to be implicated in vesicle formation and in uptake of lipid signalling molecules. Pairs with TMEM30A ($\beta$ subunit), a putative receptor of the BBB-crossing single-domain antibody FC5 (van der Mark *et al.* 2014; Abulrob *et al.* 2007) |
| Early endosome antigen 1 | Eea1 | 11% | 89% | Binds phospholipid vesicles containing phosphatidylinositol 3-phosphate and participates in endosomal trafficking (Simonsen *et al.* 1998) |
| Clathrin heavy chain 1 | Cltc | 12% | 88% | Clathrin is the major protein of the polyhedral coat of coated pits and vesicles. Plays a role in early autophagosome formation (Ravikumar *et al.* 2010; Amaya *et al.* 2015) |
| Ras-related protein Rab-5A | Rab5a | 17% | 83% | The small GTPases Rab are key regulators of intracellular membrane trafficking, from the formation of transport vesicles to their fusion with membranes. Rabs cycle between an inactive GDP-bound form and an active GTP-bound form that is able to recruit to membranes different sets of downstream effectors directly responsible for vesicle formation, movement, tethering and fusion. RAB5A is required for the fusion of plasma membranes and early endosomes (Hoffenberg *et al.* 2000). |
| Prolow-density lipoprotein receptor-related protein | Lrp1 | 20% | 80% | Receptor involved in endocytosis and in phagocytosis of apoptotic cells. Involved in cellular lipid homeostasis. Involved in the plasma clearance of activated LRPAP1 (alpha 2-macroglobulin), as well as the local metabolism of complexes between |

| | | | | |
|---|---|---|---|---|
| 1 | | | | plasminogen activators and their endogenous inhibitors. Necessary for the alpha 2-macroglobulin-mediated clearance of secreted amyloid precursor protein and beta-amyloid (Kinoshita *et al.* 2003). Implicated in BBB receptor-mediated transport of peptide ligands (Aprotinin, Angiopep) (Demeule *et al.* 2008) |
| Cell cycle control protein 50A | Tmem30a | 20% | 80% | Accessory component of a P4-ATPase flippase (see above). Putative receptor of the BBB-crossing single-domain antibody FC5 (van der Mark *et al.* 2014; Abulrob *et al.* 2007) |
| Cellubrevin | Vamp3 | 31% | 69% | SNARE involved in vesicular transport from the late endosomes to the trans-Golgi network. May play a role in synaptic vesicle transport and membrane fusion (Hegedus *et al.* 2013; Han *et al.* 2017) |
| Insulin receptor | Insr | 36% | 64% | Receptor tyrosine kinase which mediates the pleiotropic actions of insulin. Binding of insulin leads to phosphorylation of several intracellular substrates. Phosphorylation of IRSs proteins leads to the activation of two main signaling pathways: the PI3K-AKT/PKB pathway, which is responsible for most of the metabolic actions of insulin, and the Ras-MAPK pathway, which regulates expression of some genes and cooperates with the PI3K pathway to control cell growth and differentiation. In addition to binding insulin, the insulin receptor can bind insulin-like growth factors (IGFI and IGFII). An antibody against insulin receptor (Boado and Pardridge 2017) is in clinical trials as brain delivery 'carrier' via receptor-mediated transport. |
| Insulin-like growth factor 1 receptor | Igf1r | 42% | 58% | Receptor tyrosine kinase which mediates actions of insulin-like growth factor 1 (IGF1). Binds IGF1 with high affinity and IGF2 and insulin (INS) with a lower affinity. The activated IGF1R is involved in cell growth and survival control. Ligand binding activates the receptor kinase, leading to receptor autophosphorylation, and tyrosines phosphorylation of multiple substrates, and subsequent activation of two main signalling pathways: the PI3K-AKT/PKB pathway and the Ras-MAPK pathway. Single-domain antibodies against IGF1R which do not activate signalling have been shown to cross the BBB via receptor-mediated transport and are being |

| | | | | |
|---|---|---|---|---|
| | | | | developed as BBB ‘carriers’ for various cargos (Stanimirovic *et al.* 2017). |
| | | | | |
| Flotillin-2 | Flot2 | 54% | 46% | May play a role in axon growth and regeneration. May be involved in epidermal cell adhesion and epidermal structure and function. Colocalized with activated GPI-linked cell adhesion molecules at the plasma membrane where transmembrane signalling may°Ccur (Bodin *et al.* 2014) |
| CD63 antigen | Cd63 | 60% | 40% | Functions as cell surface receptor for TIMP1 and plays a role in the activation of cellular signalling cascades. Plays a role in the activation of ITGB1 and integrin signalling, leading to the activation of AKT, FAK/PTK2 and MAP kinases. Promotes cell survival, reorganization of the actin cytoskeleton, cell adhesion, spreading and migration, via its role in the activation of AKT and FAK/PTK2. Plays a role in VEGFA signalling via its role in regulating the internalization of KDR/VEGFR2. Plays a role in intracellular vesicular transport processes, and is required for normal trafficking of the PMEL luminal domain that is essential for the development and maturation of melanocytes. Plays a role in the adhesion of leukocytes onto endothelial cells via its role in the regulation of SELP trafficking (Doyle *et al.* 2011). |
| Lysosome-associated membrane glycoprotein 1 | Lamp1 | 63% | 37% | Presents carbohydrate ligands to selectins. Also implicated in tumor cell metastasis. A lysosomal acidic membrane protein (Wang *et al.* 2017). |
| Flotillin-1 | Flot1 | 65% | 35% | May act as a scaffolding protein within caveolar membranes, functionally participating in formation of caveolae or caveolae-like vesicles. Co-localized with activated GPI-linked cell adhesion molecules at the plasma membrane where transmembrane signaling may°Ccur (Bodin *et al.* 2014) |
| Synaptobrevin | Vamp2 | 72% | 28% | Involved in the targeting and/or fusion of transport vesicles to their target membrane. Plays a role in membrane fusion in neuronal exocytosis (Han *et al.* |

| | | | | |
|---|---|---|---|---|
| | | | | 2017). |
| Caveolin-1 | Cav1 | 73% | 27% | Involved in the costimulatory signal essential for T-cell receptor (TCR)-mediated T-cell activation. May act as a scaffolding protein within caveolar membranes. Interacts directly with G- protein alpha subunits and can functionally regulate their activity. Forms a stable hetero-oligomeric complex with CAV2 that targets to lipid rafts and drives caveolae formation (Andreone *et al.* 2017) |
| Synaptosomal-associated protein 23 | Snap23 | 75% | 25% | Essential component of the high affinity receptor for the general membrane fusion machinery and an important regulator of transport vesicle docking and fusion. Participates in the formation of 20S SNARE complexes along with syntaxin 4 and VAMP2/3 (Han *et al.* 2017) |
| Cation-dependent mannose-6-phosphate receptor | M6pr | 80% | 20% | Transport of phosphorylated lysosomal enzymes from the Golgi complex and the cell surface to lysosomes. Lysosomal enzymes bearing phosphomannosyl residues bind specifically to mannose-6- phosphate receptors in the Golgi apparatus and the resulting receptor-ligand complex is transported to an acidic prelysosomal compartment where the low pH mediates the dissociation of the complex (Olson *et al.* 2008). |
| Ras-related protein Rab-7a | Rab7a | 84% | 16% | Key regulator in endo-lysosomal trafficking. Governs early-to-late endosomal maturation, microtubule minus-end as well as plus-end directed endosomal migration and positioning, and endosome-lysosome transport through different protein-protein interaction cascades. Also involved in regulation of some specialized endosomal membrane trafficking, such as maturation of melanosomes, pathogen-induced phagosomes (or vacuoles) and autophagosomes (Amaya *et al.* 2015) |
| Ras-related protein Rab-11A | Rab11a | 91% | 9% | The small GTPases Rab are key regulators of intracellular membrane trafficking, from the formation of transport vesicles to their fusion with membranes. Rabs cycle between an inactive GDP-bound form and an active GTP-bound form that is able to recruit to membranes different set of downstream effectors directly responsible for vesicle formation, movement, |

| | | | | |
|---|---|---|---|---|
| | | | | tethering and fusion. Regulates the recycling of FCGRT (receptor of Fc region of monomeric IgG) to basolateral membranes (Amaya *et al.* 2015) |

**Description of SV-ARBEC cell line**.

SV-ARBEC line was derived from primary brain endothelial cells isolated as described (Morley *et al.* 1998) by immortalization with the Simian Virus 40 (SV-40) using protocols described in (Muruganandam *et al.* 1997), and were characterized as BBB model *in vitro* as described in (Garberg *et al.* 2005). The karyotype authentication of SV-ARBEC was performed in 2003 prior to banking. Cells were banked at passage 76 and used in these studies between passage 78 and 86. The expression of rat-specific genes/variants was confirmed using high throughput sequencing in 2017. SV-ARBEC is not listed as a commonly misidentified cell line by the International Cell Line Authentication Committee (ICLAC; http://iclac.org/databases/cross-contaminations/);

**Reference list**


Abulrob A., Stanimirovic D., Muruganandam A. (2007) *Blood-brain barrier epitopes and uses thereof. Patent application 12/890,079, USA*.

Amaya C., Fader C. M., Colombo M. I. (2015) Autophagy and proteins involved in vesicular trafficking. *FEBS Lett.* **589**, 3343–3353.

Andreone B. J., Chow B. W., Tata A., Lacoste B., Ben-Zvi A., Bullock K., Deik A. A., Ginty D. D., Clish C. B., Gu C. (2017) Blood-Brain Barrier Permeability Is Regulated by Lipid Transport-Dependent Suppression of Caveolae-Mediated Transcytosis. *Neuron* **94**, 581–594.e5.

Boado R. J., Pardridge W. M. (2017) Brain and organ uptake in the Rhesus monkey in vivo of recombinant iduronidase compared to an insulin receptor antibody-iduronidase fusion protein. *Mol. Pharm.* **14**, 1271–1277.

Bodin S., Planchon D., Rios Morris E., Comunale F., Gauthier-Rouviere C. (2014) Flotillins in intercellular adhesion - from cellular physiology to human diseases. *J. Cell Sci.* **127**, 5139–5147.

Demeule M., Currie J.-C., Bertrand Y., Ché C., Nguyen T., Régina A., Gabathuler R., Castaigne J.-P., Béliveau R. (2008) Involvement of the low-density lipoprotein receptor-related protein in the transcytosis of the brain delivery vector angiopep-2. *J. Neurochem.* **106**, 1534–44.

Doyle E. L., Ridger V., Ferraro F., Turmaine M., Saftig P., Cutler D. F. (2011) CD63 is an essential cofactor to leukocyte recruitment by endothelial P-selectin. *Blood* **118**, 4265–4273.

Garberg P., Ball M., Borg N., Cecchelli R., Fenart L., Hurst R. D., Lindmark T., et al. (2005) In vitro models for the blood-brain barrier. *Toxicol. Vitr.* **19**, 299–334.

Han J., Pluhackova K., Böckmann R. A. (2017) *The multifaceted role of SNARE proteins in membrane fusion*.

Hegedus K., Takats S., Kovacs A. L., Juhasz G. (2013) *Evolutionarily conserved role and physiological relevance of a STX17/Syx17 (syntaxin 17)-containing SNARE complex in autophagosome fusion with endosomes and lysosomes*.

Hoffenberg S., Liu X., Nikolova L., Hall H. S., Dai W., Baughn R. E., Dickey B. F., et al. (2000) A novel membrane-anchored Rab5 interacting protein required for homotypic endosome fusion. *J. Biol. Chem.* **275**, 24661–24669.

Kinoshita A., Shah T., Tangredi M. M., Strickland D. K., Hyman B. T. (2003) The Intracellular Domain of the Low Density Lipoprotein Receptor-related Protein Modulates Transactivation Mediated by Amyloid Precursor Protein and Fe65. *J. Biol. Chem.* **278**, 41182–41188.

Mark V. A. van der, Waart D. R. de, Ho-Mok K. S., Tabbers M. M., Voogt H. W., Oude Elferink R. P. J., Knisely A. S., Paulusma C. C. (2014) The lipid flippase heterodimer ATP8B1–CDC50A is essential for surface expression of the apical sodium-dependent bile acid transporter (SLC10A2/ASBT) in intestinal Caco-2 cells. *Biochim. Biophys. Acta - Mol. Basis Dis.* **1842**, 2378–2386.

Morley P., Small D. L., Murray C. L., Mealing G. A., Poulter M. O., Durkin J. P., Stanimirovic D. B. (1998) Evidence that Functional Glutamate Receptors are not Expressed on Rat or Human Cerebromicrovascular Endothelial Cells. *J. Cereb. Blood Flow Metab.* **18**, 396–406.

Muruganandam A., Herx L. M., Monette R., Durkin J. P., Stanimirovic D. B. (1997) Development of immortalized human cerebromicrovascular endothelial cell line as an in vitro model of the human blood-brain barrier. *FASEB J.* **11**, 1187–97.

Olson L. J., Hindsgaul O., Dahms N. M., Kim J.-J. P. (2008) Structural Insights into the Mechanism of pH-dependent Ligand Binding and Release by the Cation-dependent Mannose 6-Phosphate Receptor. *J. Biol. Chem.* **283**, 10124–10134.

Ravikumar B., Moreau K., Rubinsztein D. C. (2010) Plasma membrane helps autophagosomes grow. *Autophagy* **6**, 1184–1186.

Simonsen A., Roger L., Christoforidis S., Gaullier J.-M., Brech A., Callaghan J., Toh B.-H., Murphy C., Zerial M., Stenmark H. (1998) EEA1linksPI(3)K function toRab5regulation ofendosomefusion. *Nature* **394**, 494–498.

Stanimirovic D. B., Kemmerich K., Haqqani A. S., Sulea T., Arbabi-Ghahroudi M., Massie B., Gilbert R. (2017) *Insulin-like growth factor 1 receptor -specific antibodies and uses thereof. Patent applications: US 15/123,735, US 15/123,781, US 15/123,798.*

Wang Q., Yao J., Jin Q., Wang X., Zhu H., Huang F., Wang W., Qiang J., Ni Q. (2017) LAMP1 expression is associated with poor prognosis in breast cancer. *Oncol. Lett.* **14**, 4729–4735.
